# Determinants of adverse birth outcome in Sub-Saharan Africa: analysis of recent demographic and health surveys

**DOI:** 10.1186/s12889-021-11113-z

**Published:** 2021-06-07

**Authors:** Koku Sisay Tamirat, Malede Mequanent Sisay, Getayeneh Antehunegn Tesema, Zemenu Tadesse Tessema

**Affiliations:** Department of Epidemiology and Biostatistics, College of Medicine and Health Sciences, Institute of Public Health, University of Gondar, Gondar, Ethiopia

**Keywords:** Adverse birth outcomes, Determinants, Sub-Saharan Africa

## Abstract

**Background:**

More than 75% of neonatal deaths occurred in the first weeks of life as a result of adverse birth outcomes. Low birth weight, preterm births are associated with a variety of acute and long-term complications. In Sub-Saharan Africa, there is insufficient evidence of adverse birth outcomes. Hence, this study aimed to determine the pooled prevalence and determinants of adverse birth outcomes in Sub-Saharan Africa.

**Method:**

Data of this study were obtained from a cross-sectional survey of the most recent Demographic and Health Surveys (DHS) of ten Sub-African (SSA) countries. A total of 76,853 children born five years preceding the survey were included in the final analysis. A Generalized Linear Mixed Models (GLMM) were fitted and an adjusted odds ratio (AOR) with a 95% Confidence Interval (CI) was computed to declare statistically significant determinants of adverse birth outcomes.

**Result:**

The pooled prevalence of adverse birth outcomes were 29.7% (95% CI: 29.4 to 30.03). Female child (AOR = 0.94, 95%CI: 0.91 0.97), women attended secondary level of education (AOR = 0.87, 95%CI: 0.82 0.92), middle (AOR = 0.94,95%CI: 0.90 0.98) and rich socioeconomic status (AOR = 0.94, 95%CI: 0.90 0.99), intimate-partner physical violence (beating) (AOR = 1.18, 95%CI: 1.14 1.22), big problems of long-distance travel (AOR = 1.08, 95%CI: 1.04 1.11), antenatal care follow-ups (AOR = 0.86, 95%CI: 0.83 0.86), multiparty (AOR = 0.88, 95%CI: 0.84 0.91), twin births (AOR = 2.89, 95%CI: 2.67 3.14), and lack of women involvement in healthcare decision-making process (AOR = 1.10, 95%CI: 1.06 1.13) were determinants of adverse birth outcomes.

**Conclusion:**

This study showed that the magnitude of adverse birth outcomes was high, abnormal baby size and preterm births were the most common adverse birth outcomes. This finding suggests that encouraging antenatal care follow-ups and socio-economic conditions of women are essential. Moreover, special attention should be given to multiple pregnancies, improving healthcare accessibilities to rural areas, and women’s involvement in healthcare decision-making.

**Supplementary Information:**

The online version contains supplementary material available at 10.1186/s12889-021-11113-z.

## Background

According to the global report, about 2.9 million babies die in the first month of life, of which preterm births, complications during pregnancy, and sepsis are the leading causes of death [1–3]. Particularly, adverse birth outcomes contributed to more than 75% of neonatal deaths occurred in the first weeks of life [1]. Adverse birth outcomes are defined by the World Health Organization as events of low birth weight, preterm birth, stillbirth, or perinatal deaths [4–7]. In particular, low birth weight (LBW) is often defined as a birth weight of below 2500 g, which might be resulted from intrauterine growth retardations or shorter gestational age. Globally, about 15 to 20% of births weighted below 2500 g and associated with various neonatal health complications like hypothermia, hypoglycemia, and early deaths. Moreover, neurocognitive problems and developmental delays are the long-term complications of LBW that determine child survival and future health [4–12].

On top of that, about 15 million babies are born too preterm (often before 37 completed weeks of gestation) each year, of whom more than one million died immediately after birth due to complications and lack of appropriate treatment [13]. Many of the survivors face a lifetime of disability, including learning disabilities and visual and hearing problems [12]. Meanwhile, experiencing a stillbirth during pregnancy or childbirth is a tragedy insufficiently addressed in global agendas, policies, and funded programs.

Approximately, 2.6 million stillbirths occurred each year, of which about 50% of the incidents occurred just after the onset of labor [7]. About 84% of all stillbirths occur in low-and-middle-income countries including SSA where maternal health service coverage is low. Furthermore, stillbirth has psychological costs to women and their families, such as maternal depression, financial and economic repercussions, as well as stigma and taboo.

**Fig. 1:**
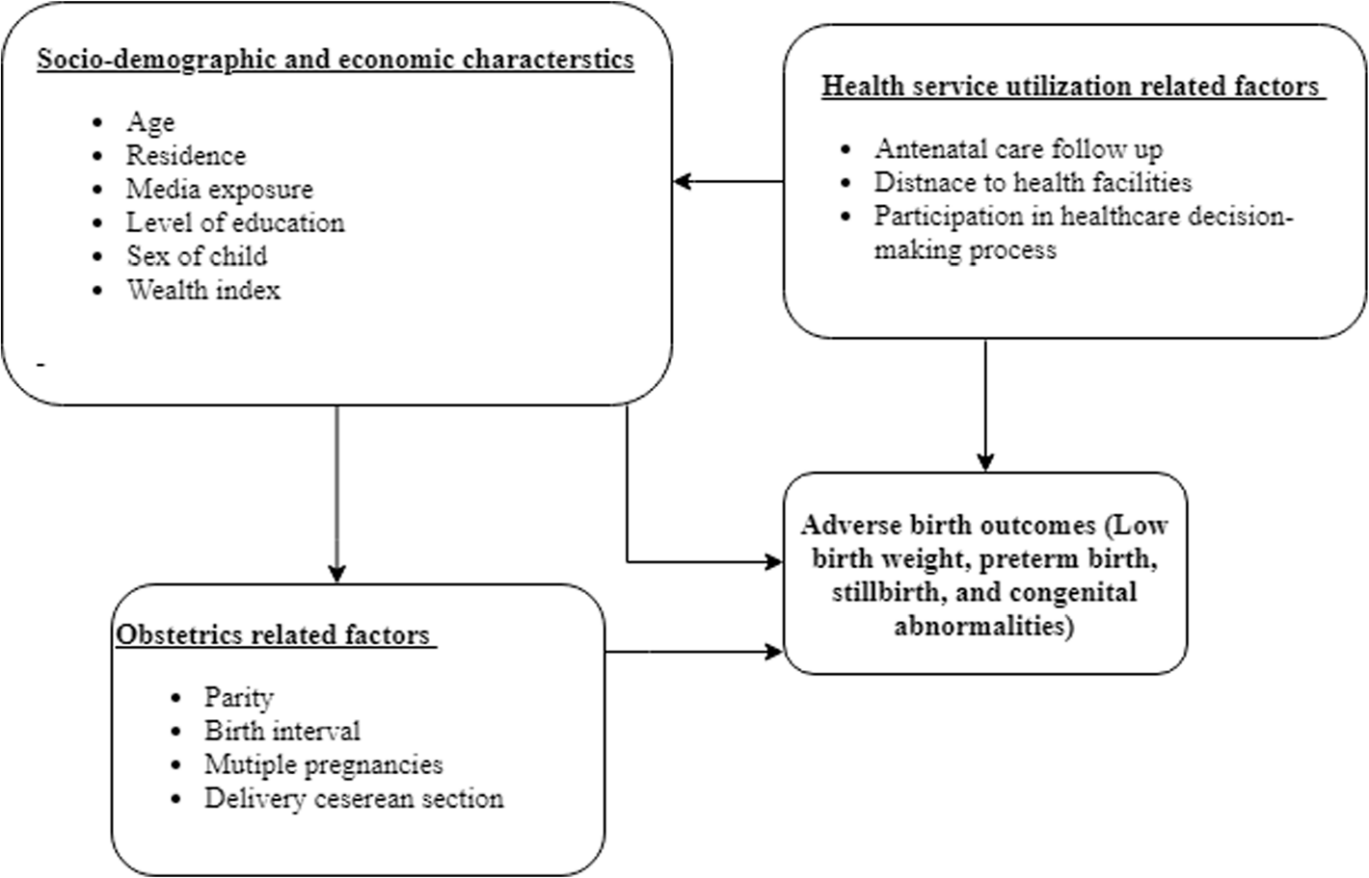
Conceptual framework of factors associated with adverse birth outcomes

A variety of intertwined maternal, nutritional, environmental, and healthcare system factors contributed to the occurrence of adverse birth outcomes. Thus, advanced maternal age, level of education, antenatal care, home delivery, and healthcare access problems, and economic conditions, maternal clinical conditions like anemia, malaria, chronic illnesses, and HIV were determinants of adverse birth outcomes (Fig. 1) [13–20].

The government and stakeholders made tremendous efforts to curb the magnitude and impacts of adverse birth outcomes. Furthermore, the reduction of adverse birth outcomes like LBW, stillbirth, and preterm births are the parts of the Sustainable Development Goal (SDG) targeted under goals 3.1 and 3.2 [21]. Focused antenatal care, institutional delivery, nutritional counseling, and improving healthcare services availability and accessibility were some of the interventions used to reduce unfavorable birth outcomes in low-income settings. For instance, continuity of maternal healthcare services led by midwifery contributed to the reduction of preterm births by 24% witnessed by previous study results [22].

Most of the studies conducted previously had been single-centered and facility-based which was less likely to be representative. In addition, there is a need for quality, large, and population based-studies from SSA countries where a large share of adverse birth outcomes occurred. Hence, this study aimed to assess the pooled prevalence and determinants of adverse birth outcomes in SSA. A better understanding of the adverse birth outcomes could provide more generalizable evidence to justify the better quality of maternal healthcare and improving accessibility of health services in low-income countries. Moreover, evidence from this study could be helpful to design and integrated efforts at the regional level to hasten favorable birth outcomes.

## Method

The most recent Demographic and Health Surveys (DHS) of ten Sub-African (SSA) countries (Angola, Congo, Cote d’Ivoire, Gambia, Lesotho, Liberia, Madagascar, Nigeria, Rwanda, Togo) data were used to make analysis of this study. The DHS is a part of the measure DHS programs that collect national information on basic health measures such as mortality, morbidity, and maternal and child health service utilization. Using the Kids Record (KR file) dataset, all births in the preceding five years before the survey were the study population. In the selected enumeration areas (EAs) births that had data about birth weight, gestational age at birth, and perinatal death records were included in the study. During the measure DHS survey, a multi-stage (two-stage) stratified sampling technique was used to select study participants; children were nested within the enumeration areas. After the dataset was appended, the weighted sample size became 76,853 children and women who had given birth five years preceding the survey. The methodology section of the DHS report goes into great detail about the study participant selection and data collection [23].

### Measurements

The main outcome variable of this study was adverse birth outcomes, which is defined as the presence of at least one or more of the following conditions in recent pregnancy (low birth weight, macrosomia, preterm birth, or stillbirth) [13, 19]. The outcome variable was generated by composite low birth weight, macrosomia, stillbirth, and gestational age less than 37 weeks of pregnancy. Finally, the variable takes 1 if at least one of adverse birth outcomes reported which was labeled as “adverse birth outcome”, and 0 otherwise.

### Independent variables

Socio-demographic characteristics (residence, maternal education, husband education, maternal age, mother marital status, sex of the child, media exposure, household wealth index, and maternal working status), health service utilization and accessibility (women healthcare decision-making autonomy, ANC follow up, and distance to health facility), and obstetrics related characteristics (preceding birth interval, parity, type of birth, and delivery by CS) were explanatory variables identified after thorough review of literatures [13, 24–31] .

Short birth interval is defined as the time between two births which is less than 24 months [32]. Also, women’s healthcare decision-making autonomy is the ability of the women to make decisions to use health care services and treatment options [25]. Finally, media exposure was defined as when a woman reads a newspaper or listens to the radio, or watches television at least three times per week.

### Data management and analysis

Before any statistical analysis, the data were weighted using sampling weight based on primary sampling unit, and strata to restore the representativeness of the survey and take sampling design when calculating standard errors and reliable estimates. Cross-tabulations and summary statistics were done using STATA software version 14 (StataCorp.2015. Stata Statistical Software: Release 14. College Station, TX: StataCorp LP). The pooled prevalence of adverse birth outcomes with a 95% Confidence Interval (CI) was reported using a forest plot. The DHS dataset has a hierarchical structure that failed to meet the standard logistic regression model assumptions of independent observation and equal variance. Meanwhile, the children were nested within a cluster household, and children from the same cluster were more similar than from other clusters. Therefore, a mixed effect logistic regression model (both fixed and random effect) was fitted to account for cluster variability by using the advanced models. The outcome variable of the study was binary, a standard logistic regression and Generalized Linear Mixed Models (GLMM) were fitted step by step. Because the models were nested, model fitness was compared using the Intra-class Correlation Coefficient (ICC), Likelihood Ratio (LR), Median Odds Ratio (MOR), and deviance (−2LLR) values. As a result, the mixed-effect logistic regression model with the lowest deviance value was selected as the most parsimonious model. (Shown on Supplementary file Table 1). In the bivariable analysis, variables with less than 0.2 *p*-values were selected and entered into the multivariable mixed-effect logistic regression model. Adjusted Odds Ratios (AOR) with a 95% CI were calculated in the multivariable model to see the strength of association between independent variables and adverse birth outcomes. Variables with a 0.05 p-value in the final model being used as a statistically significant determinant of adverse birth outcomes.

### Ethical consideration

Permission for data access was obtained from measure demographic and health survey through an online request from http://www.dhsprogram.com. The data used for this study were publicly available with no personal identifier.

## Result

### Socio-demographic characteristics

The median age of the women in this sample was 28 years, with an interquartile range of 24 to 28 years, and half of them (51.1%) were between the ages of 20 and 29 years. Nearly two-thirds of mothers (62.5%) lived in rural areas, 19.7% had no formal schooling, and 83.8% were married at the time of data collection. Whilst more than one-third (34.3%) of women experienced physical violence (beating) from the intimate partner due to the reason of refusals for sex, neglect of a child, and goes out of home without telling to the husband (Table 1).

**Table 1:** The socio-demographic and economic characteristics of the study population in Sub-Saharan Africa

Variables	Weighted frequency	Percentage (%)
**Country**		
Angola	7154	9.3
Congo	10,663	13.9
Côte d’Ivoire	2105	2.7
Gambia	14,605	19
Lesetho	10,415	13.6
Liberia	7783	10.1
Madagascar	5201	6.8
Nigeria	3081	4
Rwanda	8156	10.6
Togo	7690	10
**Residence**		
Urban	28,825	37.5
Rural	48,028	62.5
**Maternal age**		
15–19	4385	5.7
20–29	39,255	51.1
30–39	27,325	35.5
40–49	5878	7.7
**Mothers education status**		
No	15,158	19.7
Primary	31,832	41.4
Secondary and above	29,863	38.9
**Husband education status**		
No	9921	12.9
Primary	22,977	29.9
Secondary and above	43,955	57.2
**Marital status**		
Married	64,416	83.8
Divorced/widowed/not living together	12,437	16.2
**Wealth status**		
Poor	27,538	35.8
Middle	15,470	20.1
Rich	33,845	44.1
**Woman in paid employment**		
No	24,789	32.3
Yes	52,064	67.7
**Women involvement in healthcare decision-making**		
No	45,304	58.9
Yes	31,549	41.1
**Media exposure**		
No	51,947	67.6
Yes	24,906	32.4
**Experienced intimate partner beating**		
Yes	26,391	34.3
No	50,462	65.7

### Obstetrics characteristics of women in SSA

About 71.4% of women had antenatal care follow up for the recent pregnancies, 94.2% of women gave birth in health institutions, of whom 7.6% births were by cesarean section mode of delivery. The majority (87.7%) women had greater than 24 birth months’ of the interval from the preceding births and 15.2% were on the birth order of 6th and above (Table 2).

**Table 2:** Maternal obstetrics characteristics of the study population in Sub-Saharan Africa

Characteristics	Frequency	Percentage
**Number of ANC visits**		
No visit	21,948	28.6
1–3 visits	18,134	23.6
4+ visits	36,771	47.8
**Place of delivery**		
Home	4467	5.8
Health institution	72,386	94.2
**Mode of delivery**		
Vaginal	70,770	92.4
Cesarean section	5797	7.6
**Iron supplementation**		
Yes	48,165	85.3
No	8309	14.7
**Sex of child**		
Male	38,964	50.7
Female	37,889	49.3
**Type of pregnancy**		
Single	74,179	96.5
Twin	2674	3.5
**Birth order**		
1–2	35,618	46.4
3–5	29,518	38.4
≥ 6	11,717	15.2
**Preceding birth intervals**		
< 24	9411	12.3
≥ 24	67,442	87.7
**Ever termination of pregnancy**		
Yes	9813	12.8
No	67,040	87.2

### The pooled prevalence of the adverse birth outcome in SSA

**Fig. 2:**
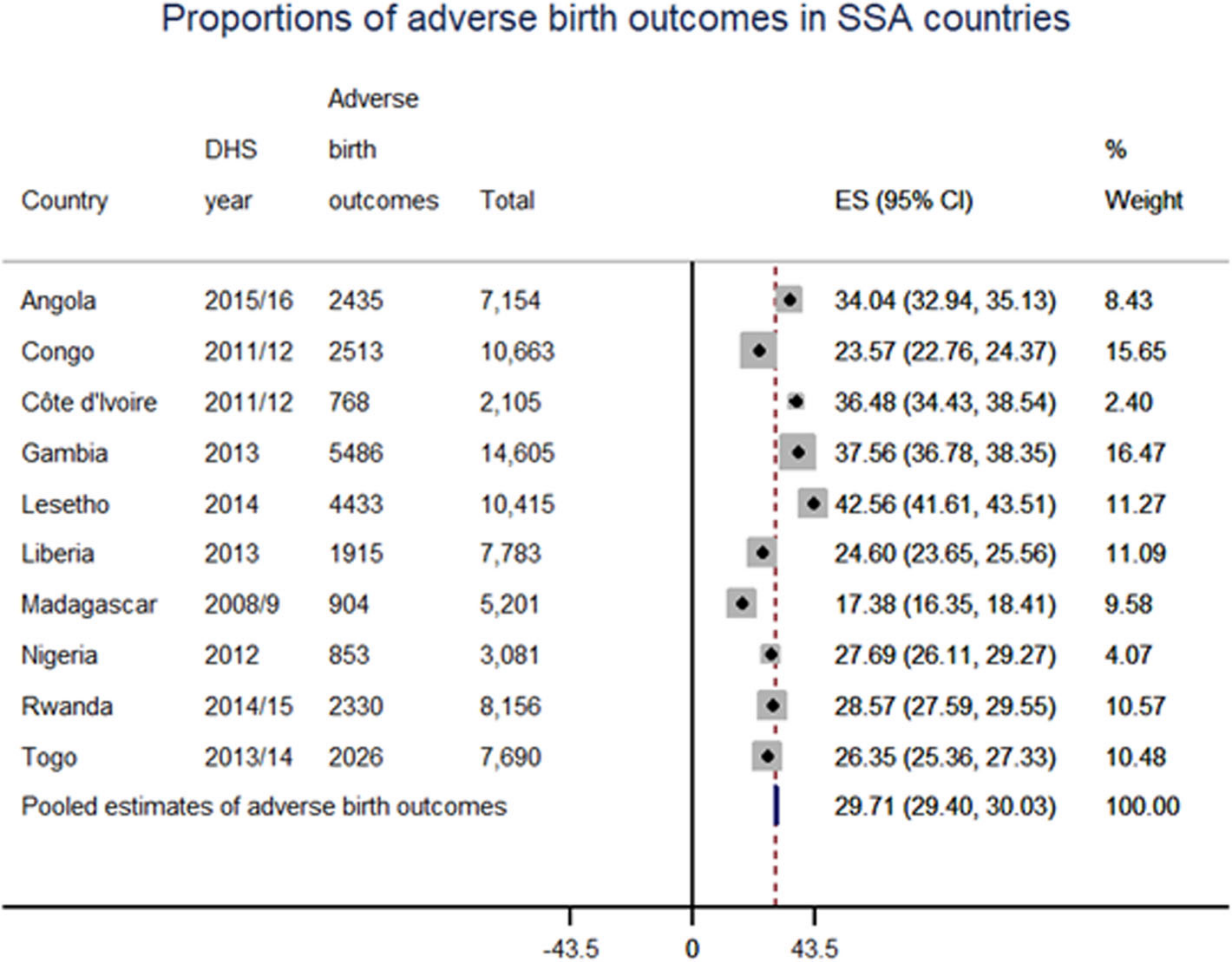
Forest plot of adverse birth outcomes in Sub-Saharan Africa countries

The prevalence of adverse birth outcomes in Sub-Saharan countries was 29.7% with a 95% CI of 29.4 to 30.03%, variations among countries also observed ranged from 17.4% in Madagascar to 42.6% in Lesotho (Fig. 2). More specifically, the rate of stillbirth was 11.5 per 1000 births; with preterm birth, fetal low birth weight, and macrosomia accounted for 7.6, 14.7, and 25.1% of all births, respectively. Of the reported adverse birth outcomes; macrosomia, low birth weight, preterm birth, and stillbirth accounted for 44, 31%, 22, and 3% of cases, respectively, and 3.24% had more than one adverse birth outcome.

### Determinants of adverse birth outcomes in SSA

In the multivariable mixed-effect logistic regression analysis, sex of a child, maternal level of education, socioeconomic status, intimate partner violence (beating), women-autonomy, distance to the health facility, parity, and twin birth were associated with adverse birth outcomes. Thus, the odds of adverse birth outcomes decreased among middle (AOR = 0.94, 95%CI: 0.90 0.98) and rich socioeconomic status (AOR = 0.90, 95%CI: 0.90 0.99) compared to poorer women. Similarly, for women who attended secondary and above levels of education, the odds of adverse birth outcomes decreased by 13% (AOR = 0.87, 95%CI: 0.82 0.92) than those who had no formal education. Likewise, antenatal care visits and multiparity birth associated with 14% (AOR = 0.86, 95%CI: 0.83 0.89) and 12% (AOR = 0.88, 95%CI: 0.84 0.91) lower odds of adverse birth outcomes compared to those who had no follow-ups and those primiparous women, respectively. In addition, female child associated with 6% (AOR = 0.94, 95%CI: 0.91 0.97) lower odds of adverse birth outcomes than males. Whilst, mothers who experienced intimate partner violence (beating) had 1.18 (AOR = 1.18, 95%CI: 1.14 1.22) times higher odds of adverse birth outcomes than women with no such history. In the same way, for women who didn’t participate in healthcare decision-making, the odds of adverse birth outcomes were 1.10 (AOR = 1.10, 95%CI: 1.06 1.13) times higher than participated in decision-making. Similarly, women who perceived distance to health facilities as big problems had 1.08 (AOR = 1.08, 95%CI: 1.04 1.11) times higher compared to the counterpart. Furthermore, twin birth was associated with 2.89 (AOR = 2.89, 95%CI: 2.67 3.14) times more likely to had adverse birth outcomes than singleton birth (Table 3).

**Table 3:** Multivariable multilevel logistic regression analysis to identify factors associated with low adverse birth outcomes in sub-Saharan Africa

Characteristics	Adverse birth outcome		Odds Ratio (OR)	
	Yes	No	Crude OR	Adjusted OR
**Maternal age**				
15–19	1478	2917	1	1
20–29	12,181	27,074	0.91 (0.85 0.98)	0.95 (0.89 1.02)
30–39	8217	19,108	0.91 (0.85 0.98)	0.98 (0.91 1.07)
40–49	1787	4091	0.86 (0.86 1.02)	0.99 (0.89 1.09)
**Sex of child**				
Male	12,215	26,749	1	1
Female	11,448	26,441	0.94 (0.91 0.97)	0.94 (0.91 0.97)*
**Residence**				
Urban	8204	20,621	1	1
Rural	15,459	32,569	1.15 (1.11 1.19)	1.02 (0.98 1.06)
**Maternal level of education**				
No formal education	4669	10,489	1	1
Primary education	10,956	20,876	1.00 (0.96 1.05)	1.04 (0.99 1.09)
Secondary and above	8038	21,825	0.80 (0.76 0.84)	0.87 (0.82 0.92)*
**Media exposure**				
Yes	7949	16,957	0.89 (0.86 0.92)	0.97 (0.94 1.01)
No	15,714	36,233	1	1
**Wealth index**				
Poor	9190	18,348	1	1
Middle	4790	10,680	0.90 (0.86 0.94)	0.94 (0.90 0.98)*
Rich	9683	24,162	0.82 (0.80 0.85)	0.92 (0.90 0.99)*
**Women didn’t participate in healthcare decision making**				
Yes	13,396	31,908	1	1
No	10,267	21,282	1.12 (1.09 1.16)	1.10 (1.06 1.13)*
**Distance to a health facility**				
Big problems	9215	17,979	1.13 (1.09 1.17)	1.08 (1.04 1.11)*
No big problem	14,448	35,211	1	1
**ANC follow up**				
Yes	7488	14,460	0.80 (0.77 0.82)	0.86 (0.83 0.89)*
No	16,175	38,730	1	1
**Parity**				
1–2 births	11,030	24,588	1	1
3–5 births	8737	20,781	093 (0.90 0.96	0.88 (0.84 0.91)*
> =6 births	3896	7821	1.07 (1.02 1.12)	0.97 (0.88 1.006)
**Birth interval**				
< 24 months	3014	6397	1	1
24 and above months	20,649	46,973	0.96 (0.91 1.00)	0.95 (0.90 1.00)
**Experienced intimate partner beating**				
Yes	8617	17,774	1.23 (1.19 1.27)	1.18 (1.14 1.22)*
No	15,046	35,416	1	1
**Type of birth**				
Single	22,198	51,981	1	
Twin	1465	1209	2.95 (2.95 3.19)	2.89 (2.67 3.14)*

* Shows a statistical significance of at 0.05 *p*-value

## Discussion

According to this report, the pooled prevalence of adverse birth outcomes was 29.7%, with (95% CI: 29.4 to 30.03%). Stillbirth rate was 11.5 per 1000 births, with preterm birth, fetal low birth weight, and macrosomia accounted for 7.6, 14.7, and 25.1% of all births, respectively. A woman’s level of education, socioeconomic status, intimate partner physical violence (beating), sex of child, women autonomy for health care decision-making, distance travel, twin birth, multiparity, and ANC follow-up were determinants of adverse birth outcomes. The rate of stillbirth in this study was lower than the results of 48.5 per 1000 births in Southeast Asia [33]. This could be because most of the stillbirths in the community are under-reported and is a common problem in SSA [34]. However, the magnitude of adverse birth outcomes in this study was higher than the study finding 18.3% in Ethiopia [19]. However, this study finding of low birth weight (14.7%%) and prematurity (7.6%) were lower than the findings of LBW (19.6%) and prematurity (17.7%) among HIV-infected women in Uganda [20]. This could be because of chronic diseases and HIV/AIDS, in which highly active antiretroviral therapy (HAART) is associated with small gestational age and preterm labor among pregnant women [35]. Similarly, this study finding of low birth weight (7.99%) and prematurity (7.60%) was lower than the previous study finds of 12.36 and 8.28% among teenagers in the US [36]. This could be due to study population differences in teen ages associated with a higher risk of adverse birth outcomes.

Various maternal and contextual factors are associated with adverse birth outcomes, thus distance travel to health facilities associated with higher odds of adverse birth outcomes. This finding was consistent with those of studies in Africa [19, 24, 32, 37, 38]. This could be due to the fact long-distance travel restricts the utilization of basic maternal health services like ANC checkups and institutional delivery. Meanwhile, long-distance travel to health facilities also directly responsible for perinatal deaths from fetal distresses [39]. Some countries like Ethiopia established maternal waiting areas (MWA) for mothers who came from hard-to-reach areas [40]. Moreover, integrating emergency obstetrics transportation with complementary maternal health services avert adverse pregnancy outcomes and improves access to skilled obstetric services for women in LMICs [41]. Likewise, women experienced intimate partner physical violence (beating) associated with increased occurrence of adverse birth outcomes. This finding was consistent with previous study findings [42].

In contrast, birth interval above 24 months is associated with a decreased risk of adverse birth outcomes. This finding was consistent with those of studies SSA [15, 35, 43–45]. This could be due to the fact associated with gestational diabetes mellitus, and a narrow inter-pregnancy interval related to lower weight losses. In addition, female child associated with lower odds of adverse birth outcomes than males. This could be due to the differential effects of stress on female and male pregnancies which are supported by previous evidence [46].

This study also revealed that twin pregnancies were associated with increased risks of adverse birth outcomes. This finding was in agreement with previous studies [31, 47]. This could be due to the reason for the high rate of pre-eclampsia and antepartum hemorrhages during pregnancies that lead to unfavorable birth outcomes. Thus, a twin pregnancy is defined as a high-risk pregnancy with special attention, and birth preparedness and complication readiness counseling should be given to pregnant mothers. Furthermore, the study’s findings point to the value of affordable imaging technology in low-income countries.

Likewise, women who didn’t participate in healthcare decision-making were more likely to had adverse birth outcomes than participated in decision makings [27]. This might be due to the reason that women declined and less involvement in healthcare decision-making was associated with decreased utilization of antennal care and institutional delivery. Moreover, a woman decreased involvement in decision-making may indicate that she is exposed to intimate partner violence [27].

On the other hand, women who had antenatal care visits for the recent birth were associated with lower odds of adverse birth outcomes than those who had no follow-up visits. This finding was consistent with those of studies in SSA [18, 19, 27, 32, 37, 45]. Antenatal care checkups help to identify most at-risk pregnancies like intrauterine growth retardation, nutritional counseling, and supplementation of nutrient fortified foods. In addition, antenatal care visits allowed identifying diseases like HIV/AIDS, syphilis, malaria, and intestinal helminthiasis infection that could affect fetal outcomes. Therefore, further improvement of quality of antenatal care and mobilization of pregnant women to WHO recommended focused care would halt adverse birth outcomes and achievement of Sustainable development goals.

Similarly, better socio-economic attributes like a better level of education and wealthy economic conditions associated with a lower risk of adverse birth outcomes. This finding was consist of previous results [15, 35, 48]. Better socio-economic attributes might hasten health-seeking behaviors and maternal nutrition. In addition, it might be associated with good knowledge of danger signs of pregnancies and early initiation of antenatal follow-ups.

This research has implications for mothers, healthcare planners, and maternal health program coordinators who are working to develop evidence-based approaches to help achieve the Sustainable Development Goals (SDG). Furthermore, increasing healthcare access to rural and hard-to-reach areas and encouraging women’s involvement in healthcare decision-making could help to minimize the extent of adverse birth outcomes. Furthermore, healthcare providers should pay more time and attention to women who had a history of partner physical violence, multiple pregnancies and have difficult labors.

This study has the strengths of a large sample size from multiple countries that would help to assess the regional situation of adverse birth outcomes. In addition, factors identified from these pooled studies might be used areas interventions for stakeholders. However, due to reasons of secondary data sources, some important clinical parameters like gestational diabetes and chronic disease conditions were not assessed. In addition, this study included only a few countries from the Sub-Saharan region that leads to under representations. Moreover, adverse birth outcomes reported in this study were based on the recent pregnancies from the Demography and health surveys.

## Conclusion

This study showed that the magnitude of adverse birth outcomes was high, abnormal baby size and preterm births were the most common types. Problems of adverse birth outcomes were more common among women who experienced intimate partner physical violence (beating), distance travel, multiple pregnancies, and lack of involvement in the healthcare decision-making process. According to the findings, improving maternal health services such as antenatal care is critical in reducing adverse birth outcomes. Furthermore, this research emphasizes the importance of paying particular attention to women who have had multiple births, expanding healthcare coverage to rural areas, and promoting women’s participation in the healthcare decision-making process.

## Supplementary Information

**Additional file 1: Supplementary file Table 1**: Model comparison and random effect results.

## Data Availability

The datasets used in this analysis are publicly available data from the DHS program, which can be accessed after filling out a data request form at http://www.dhsprogram.com.

## Abbreviations

ABO: Adverse Birth Outcomes
ANC: Antenatal Care
AOR: Adjusted Odds Ratio
CI: Confidence Interval
DHS: Demographic and Health Survey
EA: Enumeration Area
LBW: Low Birth Weight
LLR: Likelihood Ratio
SD: Standard Deviation
SGD: Sustainable Development Goal
SSA: Sub-Saharan Africa
USA: United States of America
