# Supplementary material for: Determinants of adverse birth outcome in Sub-Saharan Africa: analysis of recent demographic and health surveys
Source: BMC Public Health. 2021 Jun 7;21:1092. doi: 10.1186/s12889-021-11113-z (PMC8186187; doi:10.1186/s12889-021-11113-z)
Supplement: Supplementary file 1 — Additional file 1: Supplementary file Table 1: Model comparison and random effect results. [file 12889_2021_11113_MOESM1_ESM.docx]

Supplementary file table 1: Model comparison and random effect results

| **Parameter** | **Standard logistic regression** | **Mixed-effect logistic regression analysis (GLMM)** |
| --- | --- | --- |
| **LLR** | -46504.38 | -45684.173 |
| **Deviance** | 93008.761 | 91368.346 |
| **ICC** |  | 0.0021 |
| **LR-test** | LR test vs. logistic model: chibar2(01) = 1640.42 Prob >= chibar2 = 0.0000 | |
| **MOR** | 1.08 [1.05, 1.13] | |
| **Cluster variance** | 0.0069 [0.0025, 0.0169] | |

**LLR; log-likelihood ratio, ICC; Intra-class Correlation Coefficient, MOR; Median Odds Ratio, LR-test; Likelihood Ratio test*
